# On the basis of sex: male vs. female rat adenosine A_1_/A_2A_ receptor affinity

**DOI:** 10.1186/s13104-023-06346-7

**Published:** 2023-08-10

**Authors:** Helena D. Janse van Rensburg, Gisella Terre’Blanche, Mietha M. Van der Walt

**Affiliations:** 1 Centre of Excellence for Pharmaceutical Sciences, North-West University, Private Bag X6001, Potchefstroom, 2520 South Africa; 2 Department of Pharmaceutical Chemistry, School of Pharmacy, North-West University, Potchefstroom, South Africa; 3 Human Metabolomics, North-West University, Potchefstroom, South Africa

**Keywords:** Sex differences, Rat adenosine A_1_/A_2A_ receptors, Radioligand binding assays

## Abstract

**Objective:**

To ensure reproducibility in biomedical research, the biological variable sex must be reported; yet a reason for using male (instead of female) rodents is seldom given. In our search for novel adenosine receptor ligands, our research group routinely determines a test compound’s binding affinities at male Sprague-Dawley rat (r) adenosine A_1_ and A_2A_ receptors via *in vitro* radioligand binding studies. This pilot study compared the binding affinities of four adenosine receptor ligands (frequently used as reference standards) at male and female adenosine rA_1_ and rA_2A_ receptors.

**Results:**

The inhibition constant (*K*_i_) values determined using female rats correspond well to the values obtained using male rats and no markable difference could be observed in affinity and selectivity of reference standards. For example, DPCPX the selective adenosine A_1_ receptor antagonist: male rA_1_*K*_i_: 0.5 ± 0.1 nM versus female rA_1_*K*_i_: 0.5 ± 0.03 nM; male rA_2A_*K*_i_: 149 ± 23 nM versus female rA_2A_*K*_i_: 135 ± 29 nM. From the limited data at hand, we conclude that even when using female rats for *in vitro* studies without regard for the oestrous cycle, the obtained data did not vary much from their male counterparts.

**Supplementary Information:**

The online version contains supplementary material available at 10.1186/s13104-023-06346-7.

## Introduction

Since the discovery of receptor cloning and heterologous expression, novel compounds are evaluated at human receptors (the ultimate drug target); however, early *in vivo* studies are performed in rodents, generally *Mus musculus* (mice) and *Rattus norvegicus* (rats) which are common laboratory species [1, 2]. It is said that laboratory rats were already in use by 1850 and are most probably the first mammalian species bred specially for biological testing, given that a rat resembles the human body’s physiology (notably, rat and human neural networks are comparable) [2–4].

In our search for novel adenosine receptor ligands, our group determines a test compound’s binding affinities *in vitro* at rat adenosine A_1_ and A_2A_ receptors – more specifically at male rat adenosine receptors. This is also the case with other researchers studying adenosine receptors [5–7]; although, many do not state whether male or female rat brain membranes were used [8–12].

The rat is genetically well-characterized: In both humans and rats, the adenosine A_1_ receptor subtype contains 326 amino acids and amino acid sequence homology is 95%. The adenosine A_2A_ receptor subtype is the largest subtype and contains 412 amino acids in humans and 410 in rats with 82% amino acid sequence homology [1].

It is vital to account for sex as a biological variable to ensure reproducibility in biomedical research [13–15]; yet a reason for using male (instead of or in addition to female) rodents are seldom given. Indeed, most behavioral studies using rodents use male rodents only, seeing as researchers fear that hormonal changes during the oestrous cycle cause greater variability [16] (as well as increased costs) [13, 17]. Beery (2018) found that the ratio of male to female test subjects was 5:1 in neuroscience rodent studies [18]; furthermore, male-only studies seem to be increasing [19]. Seeing as only male rats are used, female rats are more often than not culled, though the justification for culling is controversial [20]. Female rodents are occasionally used because of ethical or economic reasons [16].

Behavioral studies have reported that female rodents are not more variable than male rodents across diverse biological traits [21–23]. In a meta-analysis of neuroscience studies, Becker et al. (2016) found that even when female rats are used in neuroscience experiments (without regard for their oestrous cycle), their data is not less consistent than their male counterparts [13]. As stated, similar results have been obtained for gene expression in humans versus rats [24].

It must; however, be noted that brain structure and chemistry are subject to sex differences, and so are adenosine and its receptors. For example, Yang et al. (2007) found that there are sex differences in the regulation of heart rate, body temperature, and locomotor activity caused by differences in adenosine A_1_ receptor expression [25]. Additionally, adenosine A_1_ and A_2A_ receptors regulate the severity of learning deficits that accompany attention-deficit hyperactivity disorder, and those deficits vary between the sexes [26]. Adenosine has also been implicated in differences in cocaine addiction between males and females, with an adenosine A_2A_ receptor antagonist having greater effects on motivation in females [27]. Both McIntosh et al. (2010) and Pierling et al. (2021) suggested that gonadal hormones, specifically oestrogen, modulate adenosine receptor gene expression, and thus, cause sex differences in adenosine receptor function [28, 29]. Although little is known about the effect of sex as a biological variable on adenosine signaling (since almost all research were performed on males), Borgus et al. (2019) found that the effects of sex and female oestrous cycle differences on the frequency and concentration of spontaneous adenosine release in male and female Sprague-Dawley rats are complex, and alas, not consistent from one brain region to the next [30].

Interestingly, neurological conditions such as Parkinson**’**s disease, depression, and dementia, among others (notably, adenosine receptors are associated with the potential treatment of these diseases), affect women and men differently; therefore, it is reasonable that rodent models of these diseases include both male and female subjects [13, 21].

In the drug discovery process, *in vivo* animal studies follow *in vitro* adenosine receptor affinity and selectivity determination (if a promising drug candidate is identified). Considering the latter, *in vitro* radioligand binding assays utilizing rat membranes expressing adenosine receptors are relevant. This pilot study aims to determine the *in vitro* binding affinities of four well-known adenosine receptor ligands (often used as reference standards) at rat adenosine A_1_ and A_2A_ receptors using male and female rat whole brain (expressing A_1_) and striatal (expressing A_2A_) membranes. To the best of our knowledge, we compare and document for the first time the variance in the *in vitro* binding affinity (inhibition constant (*K*_i_) values) of the reference standards at male and female rat adenosine A_1_ and A_2A_ receptors. Based on these results, we may provide evidence for the use of both male and female rats for *in vitro* testing of adenosine receptor ligands.

## Main text

### Materials and methods

All reagents and solvents were commercially available. [^3^H]-8-cylcopentyl-1,3-dipropylxanthine ([^3^H]DPCPX; specific activity 120 Ci/mmol) and 5′-N-[^3^H]-ethylcarboxamideadenosine ([^3^H]NECA; specific activity 27.1 Ci/mmol), Filter count (liquid scintillation cocktail) from PerkinElmer. Adenosine deaminase (5.9 mg protein/mL, 157 units/mg protein), N6-cyclopentyladenosine (CPA), caffeine, 8-cyclopentyl-1,3-dipropylxanthine (DPCPX) and istradefylline from Sigma-Aldrich. Whatman GF/B 25 mm diameter filters from Merck. Residual radioactivity was measured with a Packard Tri-CARB 2810 TR liquid scintillation counter.

### Membrane preparation

The North-West University Animal Care, Health and Safety Research Ethics Committee (NWU-AnimCare) approved the study and subsequent collection of tissue samples from adult male and female Sprague-Dawley rats for radioligand binding studies (application number NWU-00035-10-A5). The research was performed in accordance with the guidelines of the South African National Standard (SANS) document (The care and use of animals for scientific purposes). Sprague-Dawley rats were sourced from the NWU Vivarium (six-week-old, (193 ± 11.94 g). Rats were housed in medium poly-carbonated cages (2 rats per cage, male and female rats were housed separately) in a well-ventilated room at a temperature of 22 ± 2 °C and relative humidity of 50 ± 10% with a with 12 h light-dark cycle. Commercially available rat chow and tap water were provided *ad libitum*. Upon euthanasia by decapitation, 20 male and 20 female Sprague-Dawley rats were dissected and 10 male and 10 female whole brains (excluding brainstem and cerebellum) or 10 male and 10 female striata were collected and pooled separately based on sex and whole brain or striata. (Please note that rats were not treated prior to euthanasia.) Rat brain membranes were prepared and stored as described in literature [31]. The protein content of male and female rat whole brain and striatal membranes was determined using Bradford reagent and bovine serum albumin as reference standard [32].

### Adenosine A_1_/A_2A_ receptor radioligand binding assays

The A_1_ radioligand binding assay used either male or female rat whole brain membranes (expressing A_1_ receptor) and [^3^H]DPCPX (selective A_1_ antagonist) as radioligand [33] and, in turn, the A_2A_ assay used rat striatal membranes (expressing A_2A_ receptor) and [^3^H]NECA) (non-selective A_1_/A_2A_ agonist) as radioligand [34]. Each incubation of the A_1_ assay consisted of: (i) test compound (10 µL), (ii) 0.1 nM [^3^H]DPCPX (radioligand solution, 100 µL) and (iii) 120 µg rat whole brain membranes (based on protein content determined by Bradford protein assay) and 0.1 units/mL adenosine deaminase (membrane suspension, 890 µL) [31, 33]. Whereas, every incubation of the A_2A_ assay consisted of: (i) 120 µg rat striatal membranes (based on protein content determined by Bradford protein assay), 0.2 units/mL adenosine deaminase, 10 mM magnesium chloride (membrane suspension, 790 µL), (ii) test compound (10 µL), (iii) 50 nM CPA (100 µL) and (iv) 4 nM [^3^H]NECA (radioligand solution, 100 µL) [31, 34]. The final volume of all incubations contained 1 mL of 50 mM Tris.HCl buffer (pH 7.7, 25 °C) and 1% dimethylsulfoxide [31]. Non-specific binding of [^3^H]DPCPX and [^3^H]NECA for the A_1_ and A_2A_ assay, respectively, was defined as binding in the presence of 100 µM CPA [31, 33, 34]. Specific binding was defined as the total binding minus the non-specific binding [31].

### Data analysis

Data analysis was done using Microsoft Excel and GraphPad Prism Software. Sigmoidal dose response curves, from which half maximal inhibitory concentration (IC_50_) values were calculated, were obtained by plotting the specific binding against the logarithm of the test compounds′ concentrations. Subsequently, the IC_50_ values were used to calculate the inhibition constant (*K*_i_) values for the competitive inhibition of [^3^H]DPCPX (dissociation constant (*K*_d_) = 0.36 nM) [33] against rat whole brain membranes and [^3^H]NECA (*K*_d_ = 15.3 nM) [34] against rat striatal membranes by the test compounds using the Cheng-Prusoff equation [35]. Descriptive statistics were used to present *K*i values (nM) as the mean ± standard error of the mean (SEM), based on radioligand binding assays performed in triplicate. The p*K*_i_ values of reference compounds at male and female adenosine A_1_ and A_2A_ receptors were also compared, and a correlation coefficient was calculated. The R squared of the linear regression analysis is equal to the correlation coefficient.

## Results and discussion

Four adenosine receptor ligands frequently used as reference standards were investigated *in vitro* at rat adenosine A_1_ and A_2A_ receptor subtypes in male or female Sprague-Dawley rats’ whole brains (expressing A_1_) or striata (expressing A_2A_) using previously reported radioligand binding assays. The four reference standards include CPA, caffeine, DPCPX, and istradefylline. Before conducting the experiments, a literature search for *K*_i_ values was performed: the *K*_i_ values of the reference standards have been repeatedly determined at male rat adenosine receptors; however, no study reports using female rats (if the sex is at all reported).

Table 1 summarized the literature *K*_i_ values (male) and newly determined values using female rat whole brain (expressing A_1_) and striatal (expressing A_2A_) membranes, respectively. The *K*_i_ values determined using female rats correspond well to the values obtained using male rats. No markable difference could be observed in the affinity and selectivity (see selectivity index Table 1) of CPA, caffeine, DPCPX and istradefylline for the adenosine A_1_ and A_2A_ receptors.

**Table 1:** *K*_i_ values (nM) of reference standards at male and female rat adenosine A_1_ and A_2A_ receptors

Reference standard	*K*_i_ value ± SEM (nM)^a^				Selectivity index	
	**Male rA**_**1**_ **vs. 0.1 nM [** ^**3**^ **H]DPCPX**	**Female rA**_**1**_ **vs. 0.1 nM [** ^**3**^ **H]DPCPX**	**Male rA**_**2A**_ **vs. 4 nM [** ^**3**^ **H]NECA**	**Female rA**_**2A**_ **vs. 4 nM [** ^**3**^ **H]NECA**	**Male rA**_**2A**_ **K**_**i**_ **/rA**_**1**_ **K**_**i**_	**Female rA**_**2A**_ **K**_**i**_ **/rA**_**1**_ **K**_**i**_
CPA	6.5 ± 0.4^a^ 5 [36] 6 [37, 38] 7 [39] 8 [40] 10 [41, 42] 15 [43]	6.4 ± 0.7^a^	858 ± 155^a^ 163 [39] 331 [43] 400 [38] 557 [36]	852 ± 175^a^	132	133
Caffeine	52 800 ± 7 400^a^ 18 800 [44] 26 000 [45] 41 000 [10] 43 900 [46] 55 000 [47]	38 000 ± 5 220^a^	18 637 ± 4 331^a^ 22 000 [45] 32 500 [44] 43 000 [10]	21 947 ± 5 143^a^	0.4	0.6
DPCPX	0.5 ± 0.1^a^ 0.4 [48] 0.5 [43, 49]	0.5 ± 0.03^a^	149 ± 23^a^ 157 [44] 340 [51] 530 [43] 545 [39]	135 ± 29^a^	298	270
Istradefylline	125 ± 6^a^ 150 [50] 192 [38] 230 [44]	169 ± 10^a^	3.3 ± 0.9^a^ 1 [38] 2 [50, 52] 5 [44] 8 [46] 11 [41]	2.4 ± 0.4^a^	0.03	0.01

^a^Inhibition constant (K_i_, nM) value is presented as the mean ± standard error of the mean (SEM), radioligand binding assays performed in triplicate. Values without SEM are taken from the literature [10, 36–52]

**Fig. 1:**
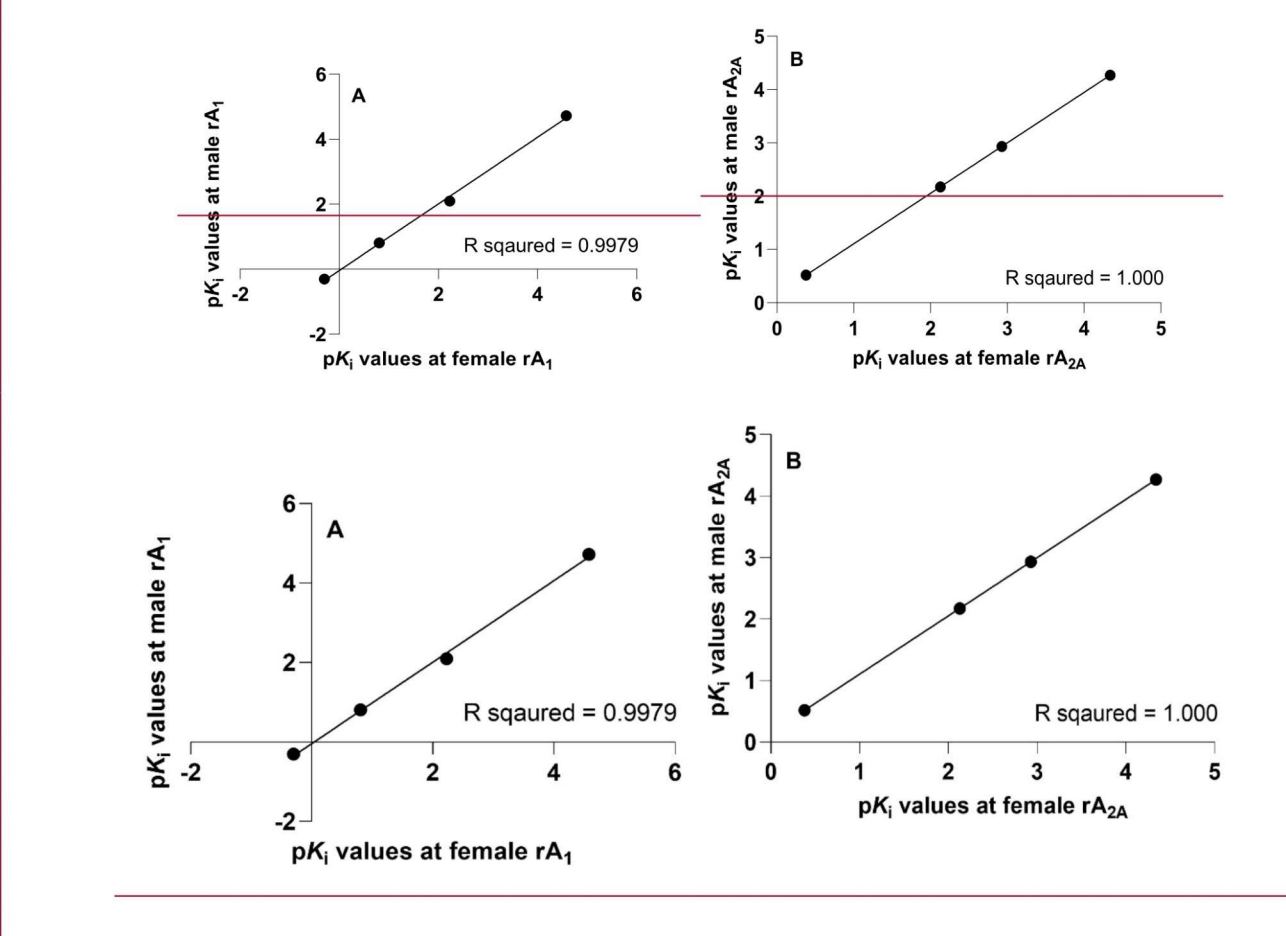
Correlation of p*K*_i_ values at male and female rat adenosine A_1_ (**A**) and A_2A_ (**B**) receptors

The p*K*_i_ values of reference compounds at male and female adenosine A_1_ and A_2A_ receptors were also compared, and a correlation coefficient was calculated. The R squared of the linear regression analysis is equal to the correlation coefficient (Fig. 1). It was found that male and female showed good correlation; with R squared values above 0.99 (i.e. >99%).

Additionally, the protein content of male and female rat whole brain and striatal membranes were almost identical as determined by a Bradford protein assay. Protein content male rA_1_: 6.91 mg/mL & rA_2A_: 6.93 mg/mL; female rA_1_: 6.13 mg/mL & rA_2A_: 6.81 mg/mL.

## Conclusion

From the limited data at hand, we conclude that even when female rats are used for *in vitro* (and not necessarily *in vivo*) studies without regard for the oestrous cycle, the obtained data is not more variable than that of their male counterparts. Indeed, the use of both male and female rats would be more ethical (by reducing the number of female Sprague-Dawley rats culled, in line with the 3 Rs: Replacement, Reduction and Refinement) as well as economical; furthermore, inclusion of both sexes in basic and preclinical research could lead to significant discoveries.

## Limitations

Although previous *in vivo* studies and the present *in vitro study* reported that female rodents are not more variable than male rodents across diverse biological traits, understanding sex differences and the influence of the female oestrous cycle is important for the design of effective treatments manipulating adenosine and its receptors. It must be noted that more standard adenosine receptor ligands should be compared to corroborate our findings, seeing as this pilot study merely presented the possibility of using female rat brain membranes for *in vitro* studies.

### Electronic supplementary material

Below is the link to the electronic supplementary material.

Supplementary Material 1

## Data Availability

The datasets used and/or analysed during the current study are available from the corresponding author on reasonable request.

## Abbreviations

r: rat
*K*_i_: inhibition constant
[^3^H]DPCPX: [^3^ H]-8-cylcopentyl-1,3-dipropylxanthine
[^3^H]NECA: 5′-N-[^3^ H]-ethylcarboxamideadenosine
CPA: N6-cyclopentyladenosine
DPCPX: 8-cyclopentyl-1,3-dipropylxanthine
NWU-AnimCare: North-West University Animal Care, Health and Safety Research Ethics Committee
SANS: South African National Standard
IC_50_: half maximal inhibitory concentration
K_d_: dissociation constant
SEM: standard error of the mean.
