# Supplementary material for: On the basis of sex: male vs. female rat adenosine A1/A2A receptor affinity
Source: BMC Res Notes. 2023 Aug 10;16:165. doi: 10.1186/s13104-023-06346-7 (PMC10413537; doi:10.1186/s13104-023-06346-7)
Supplement: Supplementary file 1 — Supplementary Material 1 [file 13104_2023_6346_MOESM1_ESM.docx]

**Figure S1.** Binding curves of reference compounds using [^3^H]DPCPX at male and female rat
whole brain membranes expressing the adenosine A_1_ receptor subtype (A) and
[^3^H]NECA at male and female rat whole brain membranes expressing the adenosine A_2A_ receptor subtype (B).
